# High-Throughput Sequencing and Exploration of the lncRNA-circRNA-miRNA-mRNA Network in Type 2 Diabetes Mellitus

**DOI:** 10.1155/2020/8162524

**Published:** 2020-05-20

**Authors:** Fang Yang, Yang Chen, Zhiqiang Xue, Yaogai Lv, Li Shen, Kexin Li, Pingping Zheng, Pan Pan, Tianyu Feng, Lina Jin, Yan Yao

**Affiliations:** ^1^Department of Health Management Center, the First Hospital of Jilin University, Jilin University, Changchun, Jilin, 130021, China; ^2^Epidemiology and Biostatistics, School of Public Health, Jilin University, Changchun, Jilin, China 130021; ^3^Department of Epidemiology and Statistics, School of Public Health, Xinjiang Medical University, Urumqi, China 830011; ^4^Department of Hospital Infection Management, Zhengzhou People's Hospital, Zhengzhou, Henan, China 450000

## Abstract

**Objective:**

Long noncoding RNA (lncRNA) and circular RNA (circRNA) are receiving increasing attention in diabetes research. However, there are still many unknown lncRNAs and circRNAs that need further study. The aim of this study is to identify new lncRNAs and circRNAs and their potential biological functions in type 2 diabetes mellitus (T2DM).

**Methods:**

RNA sequencing and differential expression analysis were used to identify the noncoding RNAs (ncRNAs) and mRNAs that were expressed abnormally between the T2DM and control groups. The competitive endogenous RNA (ceRNA) regulatory network revealed the mechanism of lncRNA and circRNA coregulating gene expression. The biological functions of lncRNA and circRNA were analyzed by Gene Ontology (GO) and Kyoto Encyclopedia of Genes and Genomes (KEGG) enrichment analysis. The candidate hub mRNAs were selected by the protein-protein interaction (PPI) network and validated by using the Gene Expression Omnibus (GEO) database.

**Results:**

Differential expression analysis results showed that 441 lncRNAs (366 upregulated and 75 downregulated), 683 circRNAs (354 upregulated and 329 downregulated), 93 miRNAs (63 upregulated and 30 downregulated), and 2923 mRNAs (1156 upregulated and 1779 downregulated) were identified as remarkably differentially expressed in the T2DM group. The ceRNA regulatory network showed that a single lncRNA and circRNA can be associated with multiple miRNAs, and then, they coregulate more mRNAs. Functional analysis showed that differentially expressed lncRNA (DElncRNA) and differentially expressed circRNA (DEcircRNA) may play important roles in the mTOR signaling pathway, lysosomal pathway, apoptosis pathway, and tuberculosis pathway. In addition, PIK3R5, AKT2, and CLTA were hub mRNAs screened out that were enriched in an important pathway by establishing the PPI network.

**Conclusions:**

This study is the first study to explore the molecular mechanisms of lncRNA and circRNA in T2DM through the ceRNA network cofounded by lncRNA and circRNA. Our study provides a novel insight into the T2DM from the ceRNA regulatory network.

## 1. Introduction

Type 2 diabetes mellitus (T2DM), formerly known as adult-onset diabetes, is a kind of non-insulin-dependent diabetes, which was one of the most common chronic diseases in the population [1]. The global prevalence of adult diabetes and impaired glucose tolerance has been increasing in recent decades [2–4]. According to the IDF Diabetes Atlas, 451 million people (aged 18 to 99 years) worldwide suffered from diabetes in 2017, and the records were predicted to augment to 693 million by 2045 [5]. Furthermore, diabetes also posed a large economic burden. The global cost of diabetes was reported to be $13100 (95% CI: 1.28-1.36) or 1.8% (95% CI: 1.8-1.9) of global gross domestic product (GDP) [6]. The occurrence and development of T2DM were mainly related to the environment and heredity. The results of the epidemiological investigation showed that obesity, high-calorie diet, and lack of physical activity were the most important environmental factors of T2DM [7]. In addition, the influence of genetic factors on T2DM has been gradually determined; for example, Gas6 gene rs8191974 and Ap3s2 gene rs2028299 were associated with T2DM in the Han population of northern China [8].

Noncoding RNA (ncRNA), discovered in recent decades, is a class of RNA molecules which has no traditional RNA function in protein translation, including long noncoding RNA (lncRNA) and circular RNA (circRNA). lncRNA is an RNA molecule with more than 200 bases in length [9], and circRNA is a kind of closed ring structure of RNA, formed by special selective shear of more than one exon [10]. Although the shapes of lncRNA and circRNA are different, they can both act as sponges of microRNA (miRNA), competing with the same corresponding miRNA response element (MRE), which can effectively control the subsequent posttranscriptional regulation of miRNA [11, 12]. Previous studies have shown that differentially expressed ncRNAs played an important role in the development of diabetes [13, 14]. For example, Ruan et al. [15] found that lncRNA-p3134 was associated with glucose metabolism and insulin signal transduction in pancreatic B cells; Zhao et al. [14] found that hsa-circ-0054633 provided a certain diagnostic ability for T2DM; Lin et al. [16] established a T2DM-related ceRNA network, extracted an mTOR-centered ceRNA subnetwork, and verified that lncRNA-NEAT1 may be associated with the mTOR signal transduction target protein mLST8 by binding to miR-181b. Nevertheless, there are few reports on RNA-mediated regulatory networks in T2DM, and there are still some shortcomings in our understanding of RNA-mediated regulatory networks. Therefore, a systematic understanding of T2DM-related RNA molecular mechanisms is essential for the development of new strategies for early diagnosis and therapeutic intervention of T2DM.

To explore the function of ncRNA in T2DM, we investigated the differential expression of lncRNA (DElncRNA), circRNA (DEcircRNA), miRNA (DEmiRNA), and mRNA (DEmRNA) by high-throughput sequencing. Blood samples were collected from three T2DM patients who were diagnosed for the first time and three healthy controls. Two competitive endogenous RNA (ceRNA) regulatory networks (the lncRNA-circRNA-miRNA-mRNA network and the protein-protein interaction (PPI) network) were further established, and the GO and KEGG enrichment analysis were performed to detect and verify the functional expression of abnormal target genes in diabetes mellitus, respectively. This is the first time that the ceRNA network established by circRNA and lncRNA has been used to explore the molecular mechanisms behind T2DM.

## 2. Materials and Methods

### 2.1. Participants

Three patients with T2DM and three age-matched healthy controls (HC) were recruited from the Second Hospital of Jilin University from August 2017 to June 2018, and all of them were all Han Chinese men aged 40-60 (Table 1). T2DM patients were diagnosed for the first time according to patients' medical history, neurological examination, and laboratory examination, and all of them had no history of using antiplatelet or antidiabetic agents. The diagnostic criteria of a T2DM patient were as follows: (1) fasting glucose (FBG) ≥ 7.0 mmol/L (fasting means at least 8 hours without calorie intake), (2) oral glucose tolerance test (OGTT) 2 h glucose ≥ 11.1 mmol/L, or (3) random blood glucose ≥ 11.1 mmol/L. Patients with a history of coronary atherosclerotic heart disease (CAD), hypertension, atrial fibrillation, myocardial infarction, tumour, acute infectious disease, immune disease, and hematological disease were excluded from the study.

### 2.2. Ethical Approval and Informed Consent

All participants provided written informed consent; the study was approved by the Ethics Committee of School of Public Health of Jilin University (ethical approval number: 2017-06-19), and it always follows the privacy of the participants.

### 2.3. Collection of Blood Samples and RNA Sequencing

The blood samples of diabetes patients and control groups were collected with a purple head anticoagulant tube in the next morning after fasting for ten hours or overnight. Total RNA was isolated and purified using RNAiso Plus (total RNA extraction reagent) (TAKARA BIO INC, CA, Japan) according to the manual. RNA purity was checked using the NanoPhotometer® spectrophotometer (IMPLEN, CA, USA). RNA concentration was measured using the Qubit® RNA Assay Kit in the Qubit® 2.0 Fluorometer (Life Technologies, CA, USA). The RNA integrity was further assessed by using the RNA Nano 6000 Assay Kit of the Agilent Bioanalyzer 2100 system (Agilent Technologies, CA, USA).

After removing the ribosomal RNA, two sequencing libraries were generated: the NEBNext® Ultra™ Directional RNA Library Prep Kit for Illumina® (NEB, USA) was used for lncRNA, circRNA, and mRNA sequencing, and the NEBNext® Multiplex Small RNA Library Prep Set for Illumina® (NEB, USA) was used for miRNA sequencing following the corresponding manufacturer's recommendations, respectively.

The clustering of the index-coded samples was performed on a cBot Cluster Generation System using TruSeq SR Cluster Kit v3-cBot-HS (Illumina) according to the manufacturer's instructions. After cluster generation, subsequent sequencings (pair-end 150 bp for circRNAs and mRNAs, single-end 50 bp for miRNAs) were conducted on the Illumina HiSeq 4000 platform (Illumina Inc.) and Illumina Hiseq 2500/2000 platform according to the manufacturer's instructions. Library construction and RNA sequencing were performed by Novogene Co., Ltd. (Beijing, China). The transcriptome data generated have been deposited in Baidu SkyDrive (https://pan.baidu.com/).

### 2.4. Quality Control of Raw Sequencing Data

To study the general characteristics and expression profiles of all DEncRNAs and DEmRNAs in the human blood sample, the RNA sequencing technology was centrally important. For lncRNA, firstly, raw data (raw reads) was obtained by removing rRNA through using an Epicentre ribo-0rRNA removal kit (Epicentre, USA) and removing free rRNA residue through ethanol precipitation. Subsequently, sequencing libraries were generated by using the rRNA-depleted RNA by NEBNext® Ultra™ Directional RNA Library Prep Kit for Illumina® (NEB, USA) following the manufacturer's recommendations. For circRNAs and mRNAs, raw data (raw reads) of fastq format was first processed through in-house perl scripts. In this step, clean data (clean reads) was obtained by removing reads containing an adapter, reads containing ploy-N, and low-quality reads from raw data. For miRNAs, raw data (raw reads) of fastq format was first processed through custom perl and python scripts. In this step, clean data (clean reads) was obtained by removing reads containing ploy-N, with 5′ adapter contaminants, without 3′ adapter or the insert tag, reads containing ploy-A, ploy-T, ploy-G, or ploy-C, and low-quality reads from raw data. At the same time, Q20, Q30, and GC-content of the all raw data were calculated. Taken together, all the downstream analyses were based on clean data.

### 2.5. RNA Sequencing Data Analysis

The significantly DElncRNAs, DEcircRNAs, DEmiRNAs, and DEmRNAs were investigated using the DESeq R package (1.8.3). A threshold value of ∣log_2_(fold change) | ≥1.5 with a *P* value < 0.05 was determined. In order to obtain an overview of the expression profiles of lncRNAs, circRNAs, miRNAs, and mRNAs, volcano plotting, heat mapping, and chromosome mapping were performed using ggplot 2, pheatmap, and karyoploteR, respectively.

### 2.6. The Construction of the ceRNA Regulatory Network

Based on the ceRNA theory, we constructed a ceRNA regulatory network for the DEncRNAs and DEmRNAs to show the regulatory relationships among lncRNA, circRNA, miRNA, and mRNA. miRanda (http://www.microrna.org/microrna) was used to predict miRNA binding seed sequence sites, and the ceRNA network which consisted of lncRNA-miRNA pairs, circRNA-miRNA pairs, and miRNA-mRNA pairs with the same miRNA nodes was visualized by Cytoscape 3.7.0.

### 2.7. Functional Analysis

To better comprehend the mechanisms of T2DM, GO analysis and KEGG pathway analysis were conducted to predict the potential functions of all DEncRNAs and DEmRNAs. Among them, GO analysis was based on three terms, namely, biological processes (BP), cellular components (CC), and molecular functions (MF), to construct gene annotation, while KEGG was a database resource for understanding high-level functions and interactions among differentially expressed genes (KEGG as a reference resource for gene and protein annotation) (http://www.genome.jp/kegg/). KOBAS software was used to test the statistical enrichment of differential expression genes in KEGG pathways.

### 2.8. The Establishment of the PPI Network and Identification of Hub mRNAs

A PPI network was constructed by STRING (https://string-db.org/) and visualized by Cytoscape 3.7.0. Then, “Molecular Complex Detection” (MCODE), a clustering algorithm identifying locally densely connected regions in a large PPI network based on node-weighting arithmetic, was employed to recognize highly interacted hub mRNA clustering.

### 2.9. Cross Validation

GSE21321, a previously published Gene Expression Omnibus (GEO) dataset including nine T2DM cases and ten normal controls, is an independent cohort which includes both miRNA and mRNA [17]. In order to validate the DEmiRNAs and DEmRNAs, we downloaded the miRNA and mRNA dataset from the GEO database. All 19 participants were Singaporean and males. Data of DEmiRNAs and DEmRNAs for GSE21321 was obtained by using GPL10322 (v.11.0 - hsa, mmu & rno [probe-level]) miRCURY LNA microRNA Array and GPL6883 (Illumina HumanRef-8 v 3.0 expression beadchip), respectively. The background correction, normalization, and summarization were performed using the robust multichip average algorithm. Then, the log_2_ transformation to the intensities of miRNAs and mRNAs was extracted from GSE21321 data. Based on these data, we used a *t*-test to perform the differential expression analyses. The individual *P* values and log_2_(fold change) were obtained to validate significance of RNAs we have found.

## 3. Result

### 3.1. Overview of the Transcriptome Profiling

Before further analyzing, we needed to take some measures to ensure the accuracy of the results. In the circRNA library, 79364942, 87375680, and 103611692 clean reads were generated in the three T2DM patients, respectively; then, 97740922, 97583408, and 100893034 clean reads were generated in the three healthy controls, respectively. In the lncRNA library, 79364942, 87375680, and 103611692 clean reads were generated in the three T2DM patients, respectively; then, 97740922, 97583408, and 100893034 clean reads were generated in the three healthy controls, respectively. The detailed quality control results are listed in Tables S1 and S2.

### 3.2. Differential Expression Analysis

**Figure 1:**
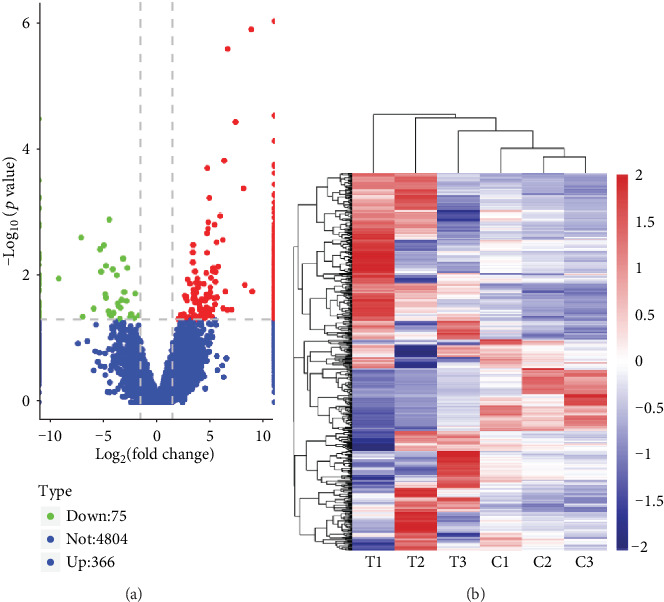
The expression profiles of lncRNAs. (a) The volcano plots of DElncRNA. Red and green indicate up- and downregulation, respectively. (b) The cluster analysis (heatmaps) of DElncRNA. The expression data was clustered with a log_10_(TPM + 1) value. The color scale indicates the expression of DElncRNAs: red and blue indicate up- and downregulation, respectively. “T” represents the T2DM samples, and “C” represents the healthy controls.

**Figure 2:**
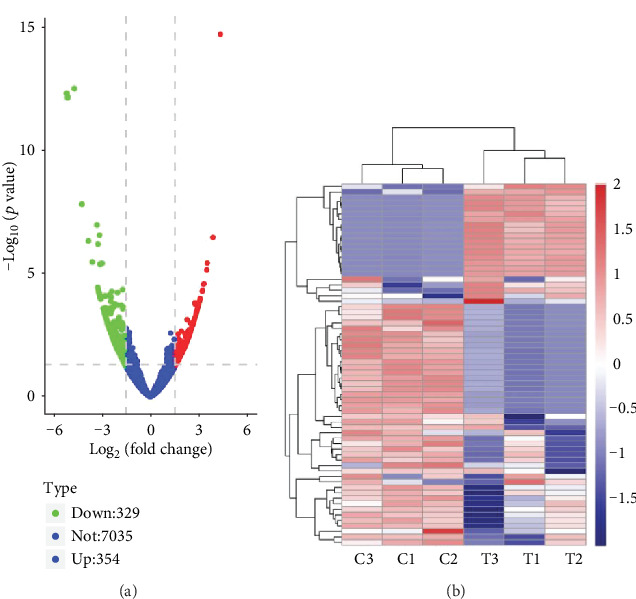
The expression profiles of circRNAs. (a) The volcano plots of DEcircRNA. Red and green indicate up- and downregulation, respectively. (b) The cluster analysis (heatmaps) of DEcircRNA. The expression data was clustered with a log_10_(TPM + 1) value. The color scale indicates the expression of DEcircRNAs: red and blue indicate up- and downregulation, respectively. “T” represents the T2DM samples, and “C” represents the healthy controls.

**Figure 3:**
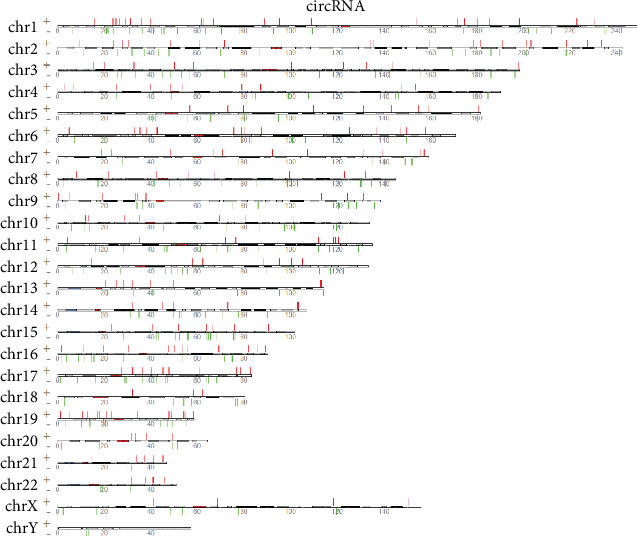
The chromosome map of DEcircRNAs between the T2DM and control groups. “+”: chromosome positive chains; “-”: chromosome negative chains; the width of the bar represents the length of the RNA.

**Figure 4:**
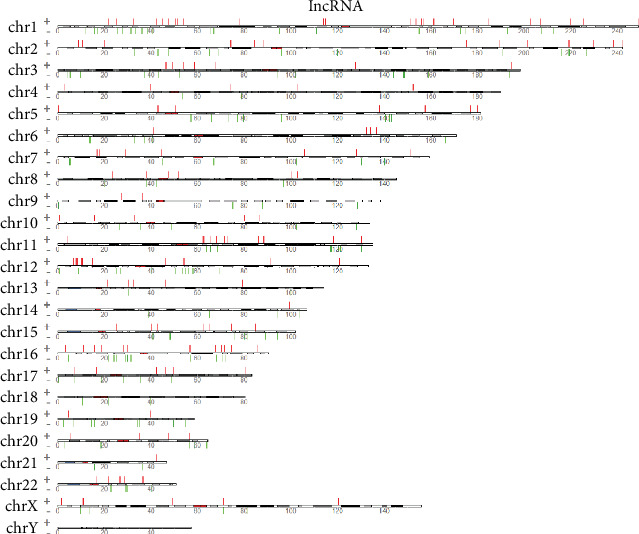
The chromosome map of DElncRNAs between the T2DM and control groups. “+”: chromosome positive chains; “-”: chromosome negative chains; the width of the bar represents the length of the RNA.

The results showed that there were clear differences in the expression profiles of lncRNAs, circRNAs, miRNAs, and mRNAs through the volcano plot (Figures 1(a)–1(b), S1A, and S2A) and heatmaps (Figures 2(a)–2(b), S1B, and S2B), respectively. In the present study, 441 lncRNAs (366 upregulated and 75 downregulated), 683 circRNAs (354 upregulated and 329 downregulated), 93 miRNAs (63 upregulated and 30 downregulated), and 2935 mRNAs (1156 upregulated and 1779 downregulated) were identified as remarkably differentially expressed in the T2DM group. The top 20 up- and downregulated lncRNAs, circRNAs, miRNAs, and mRNAs are listed in Tables S3–S6, respectively. The results also showed the position, positive and negative chains, and gene length information of the source genes of DEmRNAs (Figure S3), DEcircRNAs (Figure 3), and DElncRNAs (Figure 4) on chromosomes through the chromosome map. The chromosomes in the top five of DElncRNA distribution were as follows: chr1 (59, 13.38%), chr2 (41, 9.30%), chr12 (33, 7.48%), chr16 (29, 6.58%), and chr3 (28, 6.35%); the chromosomes in the top five of DEcircRNA distribution were as follows: chr1 (66, 9.67%), chr2 (60, 8.78%), chr12 (41, 6.00%), chr3 (40, 5.86%), and chr9 (40, 5.86%); and the chromosomes in the top five of DEmRNA distribution were as follows: chr1 (270, 9.20%), chr17 (217, 7.39%), chr19 (208, 7.09%), chr2 (177, 6.03%), and chr11 (176, 6.00%).

### 3.3. Construction of the ceRNA Regulatory Network

**Figure 5:**
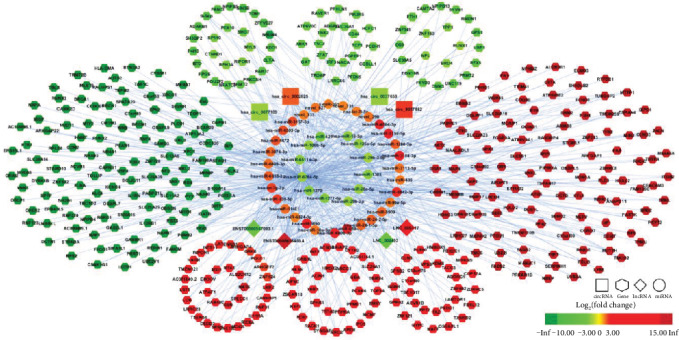
The ceRNA regulatory network of top four up- and downregulated circRNAs and lncRNAs in T2DM. In this figure, lncRNA, circRNA, miRNA, and mRNA were indicated by diamond, rectangle, ellipse, and octagon, respectively. The node color changed gradually from green to red in ascending order according to the log_2_(fold change) of RNAs.

According to the “ceRNA hypothesis,” we constructed a ceRNA regulatory network (Figure S4), by integrating the expression profiles and regulatory relationships of the lncRNAs, circRNAs, miRNAs, and mRNAs. The ceRNA regulatory network, which had more than 2 regulatory relationships, contains 364 lncRNAs (295 upregulated and 69 downregulated), 447 circRNAs (251 upregulated and 196 downregulated), 46 miRNAs (34 upregulated and 12 downregulated), and 408 mRNAs (210 upregulated and 198 downregulated). Because of the large full network, so we only showed the network of top four up- and downregulated circRNAs and lncRNAs (Figure 5).

### 3.4. GO and KEGG Pathway Analysis

**Figure 6:**
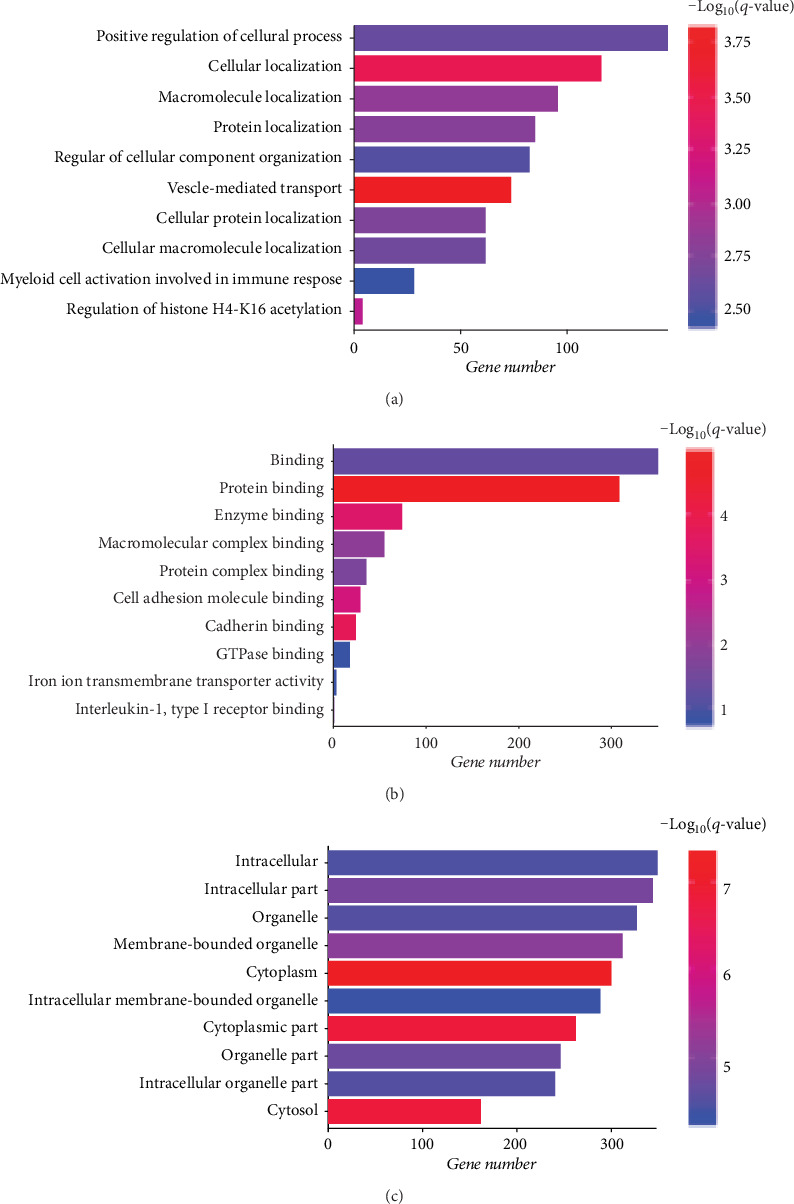
Top 10 GO enrichment annotations: biological process (a), cellular component (b), and molecular function (c). The horizontal axis stands for the gene number which was enriched on the GO term and the vertical axis for the GO term name. The node color changed gradually from blue to red in ascending order according to the negative log_10_(*q*‐value).

**Figure 7:**
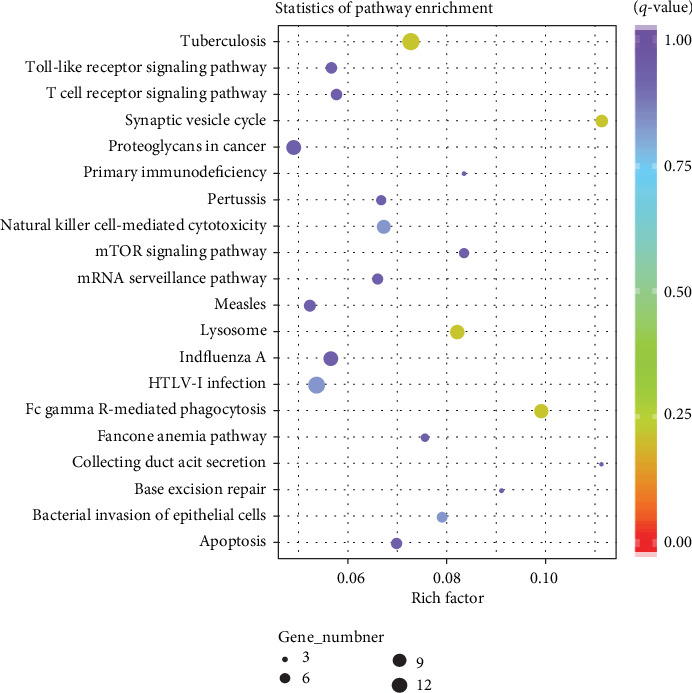
Top 20 enriched KEGG pathway analyses. The size of the spot indicated the gene numbers enriched in the pathway, and the color of the spot indicated the significance level of the enriched pathway.

To better comprehend the mechanisms involved in T2DM, we performed GO enrichment and KEGG pathway analysis. The top 10 highly enriched GO terms of BP, CC, and MF are shown in Figures 6(a)–6(c). The most enriched GO terms in BP, CC, and MF were “vesicle-mediated transport”, “cytoplasm”, and “protein binding”, respectively. According to the KEGG database, we gained 211 pathways, among which some enriched terms were involved in T2DM, such as “fc gamma R-mediated phagocytosis”, “tuberculosis”, “synaptic vesicle cycle”, “lysosome”, and “mTOR signaling pathway”. The top 20 KEGG pathways are shown in Table S7 and Figure 7, and the specific genes enriched in the top 20 KEGG pathways are shown in Table 2.

### 3.5. Identification of Hub mRNAs from the PPI Network

**Figure 8:**
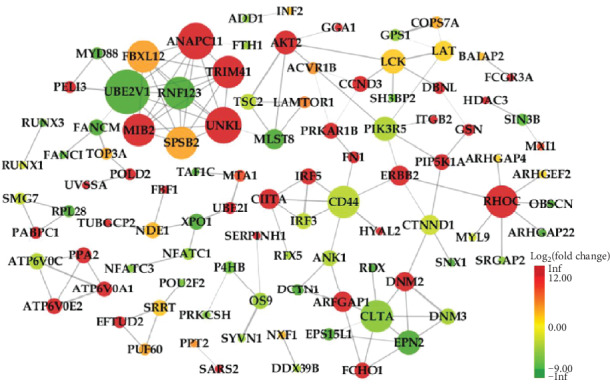
The PPI network. The node color changed gradually from green to red in ascending order according to the log_2_(fold change) of genes. The edge size changed gradually from fine to coarse in ascending order according to the combined score between two neighboring genes. The node size changed gradually from small to large in ascending order according to the degree of the node.

**Figure 9:**
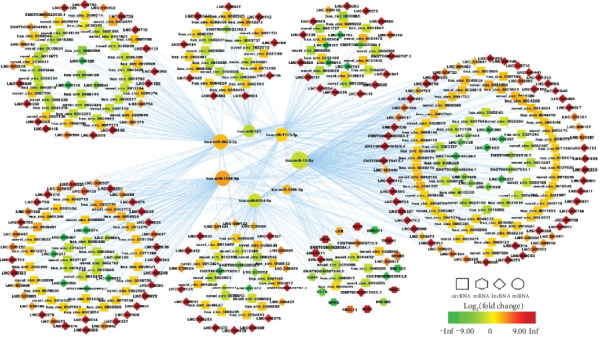
The lncRNA-circRNA-miRNA-hub mRNA network. In this figure, lncRNA, circRNA, miRNA, and mRNA were indicated by diamond, rectangle, ellipse, and octagon, respectively. The node color changed gradually from green to red in ascending order according to the log_2_(fold change) of RNAs.

To further investigate the function of genes at the protein level, we established a PPI network consisting of 100 nodes and 133 edges to view the interactions among 408 mRNAs through removing unconnected nodes (Figure 8). Considering the importance of hub mRNAs in a network, we set the mRNA with a degree greater than 5 to hub mRNAs and picked them out from the PPI network. Those fifteen mRNAs (UBE2V1, ANAPC11, UNKL, FBXL12, SPSB2, TRIM41, RNF123, MIB2, RHOC, CLTA, CD44, PIK3R5, EPN2, LCK, and AKT2) are also shown in bold in Table 2. A lncRNA-circRNA-miRNA-hub mRNA network was then built to delineate the links among the DElncRNAs, DEcircRNAs, DEmiRNAs, and DEmRNAs (Figure 9). Seven key DEmiRNAs were found from the network.

### 3.6. Validation in the GEO Dataset

The expression pattern of 93 DEmiRNAs and 15 selected DEmRNAs was verified by the GSE21321 dataset. RNA sequencing is more representative in characterizing transcripts. In this study, RNA sequencing was performed on the Illumina platform to obtain the DEmiRNAs and DEmRNAs of the T2DM. Data of DEmiRNAs and DEmRNAs for GSE21321 were obtained by using GPL10322 miRCURY LNA microRNA Array and GPL6883 Illumina HumanRef-8 v3.0 expression beadchip, respectively. However, the chips used in GSE21321 have been used early. Hence, the probes in this platform were not sufficient to detect all the DEmiRNAs and DEmRNAs in this study. Only 15 DEmiRNAs (also including has-miR-421, a key DEmiRNA) were detected in GSE21321. The *P* value and log_2_(fold change) of selected DEmiRNAs and DEmRNAs in GSE22255 are displayed in Table 3. Among them, the *P* values of the only one DEmiRNA and five DEmRNAs (has-miR-125a-5p, CD44, CLTA, UBE2V1, FBXL12, and AKT2) were all less than 0.05; that is to say, they were statistically significant. Although other 14 DEmiRNAs and 10 DEmRNAs were not significantly expressed between T2DM and normal control in GSE21321, it cannot be denied that these RNAs may play vital roles in the development of T2DM.

## 4. Discussion

With the development of molecular biotechnology, it has been found that ncRNA, which was once recognized as “noise” [18], played a crucial role in various biological processes. Recently, some studies found that the dysregulated expression of lncRNA or circRNA was related to the occurrence and development of diabetes mellitus, such as XLOC-010971, XLOC-013310 [19], and miR-7 [20]. However, there were few reports on RNA-mediated regulatory networks in diabetes, as well as reports of new transcripts. To characterize the new transcripts and biological functions of lncRNA and circRNA in T2DM, we performed high-throughput sequencing of blood samples from diabetic patients and healthy controls and transcribed and elucidated the underlying pathogenesis of T2DM.

In this study, we first performed high-throughput sequencing of blood samples from three pairs of T2DM patients and healthy controls to analyze differentially expressed lncRNA, circRNA, miRNA, and mRNA, and the results showed that there were 441 DElncRNAs and 683 DEcircRNAs. To further investigate the regulatory roles of DElncRNA and DEcircRNA in T2DM, the interaction network of lncRNA, circRNA, miRNA, and mRNA was constructed. This network showed that lncRNA and circRNA may play a central regulatory role. A single lncRNA and circRNA can be associated with multiple identical miRNAs, and then, they coregulate more mRNAs. For example, lncRNA-MIAT (the degree value is 33) and hsa-circ-0007582 (the degree value is 26) linked most miRNAs. The expression of lncRNA-MIAT was significantly higher in T2DM patients than in the control group, which was consistent with the results of Sathishkumar et al. [21]. And lncRNA-MIAT was determined to be involved in various diseases, especially myocardial infarction, diabetic retinopathy, and other microvascular complications [22], so it can be inferred that lncRNA-MIAT may also play an important role in the process of T2DM.

The biological function and potential pathways of the DElncRNAs and DEcircRNAs were initially analyzed by the GO and KEGG pathways. It was worth noting that the following significant enrichment pathways were closely related to glucose metabolism. Firstly, the mTOR signaling pathway and lysosomal pathway were closely associated with T2DM. Insulin activated mTORC1 by inducing the TSC1-TSC2-TBC1D7 complex (TSC complex) and dissociation of lysosomes [23]. Conversely, decreased activity of mTORC1 led to hypoinsulinemia [24], and excessive activation of mTORC1 led to insulin resistance [25], which has been confirmed in mouse models [26]. Secondly, apoptosis played an important role in the pathophysiology of T2DM [27], and pancreatic B cell apoptosis was a common pathological feature of T2DM. In the early stage of diabetes, B cells could overcome the lack of insulin action by increasing insulin secretion. If the function of B cells was deteriorating at this time, thus showing hyperglycemia, then the cells could not compensate for insulin resistance [28]. In T2DM, insulin resistance and visceral obesity could lead to glucose toxicity, accelerate apoptosis, and lead to B cell death [29]. However, research has shown that autophagy could play a role in resisting the cell damage caused by diabetes, protecting pancreatic *β* cells and increasing the survival rate of pancreatic *β* cells in the progression of T2DM [30]. Thirdly, the tuberculosis pathway was highly associated with diabetes. Malnutrition and lack of physical activity led to tuberculosis patients stimulating adrenaline, glucagon, and cortisol at the same time, thereby increasing glucose levels [31].

In addition, in order to further elucidate the mechanism of the ceRNA network, we also constructed a PPI network and screened fifteen hub mRNAs from the PPI network. Among them, PIK3R5, AKT2, and CLTA were mRNAs enriched in the important pathway. For example, PIK3R5 and AKT2 were mainly enriched in the mTOR signaling pathway and apoptosis pathway, while CLTA was mainly enriched in the lysosome pathway. First, the potential of PIK3R5 as a clinical biomarker for gestational diabetes mellitus has been confirmed [32]. Second, in early mouse experiments, AKT2 was proven to be an essential gene for maintaining normal glucose homeostasis [33], and it was also confirmed in subsequent studies [34]. However, there was little research on the association between CLTA and T2DM. Therefore, we can do further in-depth research on this.

Some limitations should be noted in this study. Firstly, the sample size of RNA sequencing was small, which may affect the extrapolation accuracy of the results, so it is necessary to increase the sample size to verify the results in the next step. Secondly, this is only a preliminary exploration, and the results need to be verified by experiments.

## 5. Conclusion

In conclusion, this study is the first study to explore the molecular mechanisms behind T2DM by using the ceRNA network established by circRNA and lncRNA and found that the mTOR signaling pathway, the lysosomal pathway, the apoptosis pathway, and the tuberculosis pathway were closely related to glucose metabolism. In addition, five mRNAs (CD44, CLTA, UBE2V1, FBXL12, and AKT2) were identified as the biomarkers of T2DM based on the PPI network and GEO data validation. Our study provides a novel insight into the T2DM from the ceRNA regulatory network.

## Figures and Tables

**Table 1:** Descriptive characteristics of participants.

	Case 1	Case 2	Case 3	Control 1	Control 2	Control 3
Gender	Man	Man	Man	Man	Man	Man
Race	Asian	Asian	Asian	Asian	Asian	Asian
Age	40	45	55	51	51	52
Height (cm)	172	175	173	170	172	169
Weight (kg)	98	80	70	60	76	80
DM	Yes	Yes	Yes	No	No	No
Hypertension	No	No	No	No	No	No
CAD	No	No	No	No	No	No

**Table 2:** The enriched genes in the top 20 pathways.

KEGG terms	Input genes
Fc gamma R-mediated phagocytosis	AKT2, PIK3R5, SPHK1, PIP5K1A, LAT, FCGR3A, GSN, CFL1, DNM2
Tuberculosis	CTSD, SPHK1, FCGR3A, CIITA, ITGB2, CAMK2D, MRC2, RFX5, ATP6V0C, ATP6V0A1, MYD88, HLA-DMA, AKT2
Synaptic vesicle cycle	ATP6V0E2, DNM3, ATP6V0C, NAPA, ATP6V0A1, CLTA, DNM2
Lysosome	CLN3, CTSD, PPT2, GBA, ATP6V0C, TPP1, SLC11A2, ATP6V0A1, CLTA, GGA1
Natural killer cell-mediated cytotoxicity	NFATC1, SH3BP2, LAT, ITGB2, FAS, PIK3R5, GZMB, FCGR3A, LCK
HTLV-I infection	CCND3, TCF3, CRTC1, NFATC1, NFATC3, HLA-DMA, XPO1, ITGB2, PIK3R5, CHEK2, ANAPC11, LCK, AKT2, POLD2
Bacterial invasion of epithelial cells	FN1, DNM3, SEPT9, PIK3R5, CLTA, DNM2
mTOR signaling pathway	TSC2, STRADA, PIK3R5, AKT2, MLST8
Influenza A	CIITA, HLA-DMA, XPO1, FAS, PIK3R5, DDX39B, IRF3, NXF1, MYD88, AKT2
Apoptosis	FAS, PIK3R5, PRKAR1B, MYD88, CAPN1, AKT2
Collecting duct acid secretion	ATP6V0E2, ATP6V0C, ATP6V0A1
mRNA surveillance pathway	NXF1, ACIN1, SMG7, DDX39B, PABPC1L, PABPC1
Pertussis	IRF1, MYD88, ITGB2, IRF3, CFL1
Fanconi anemia pathway	FANCM, BRCA1, FANCI, TOP3A
Base excision repair	MUTYH, POLD2, XRCC1
Proteoglycans in cancer	FN1, FLNA, ANK1, RDX, FAS, CAMK2D, PIK3R5, CD44, ERBB2, AKT2
T cell receptor signaling pathway	NFATC1, NFATC3, LAT, PIK3R5, LCK, AKT2
Toll-like receptor signaling pathway	IRF5, TOLLIP, PIK3R5, IRF3, MYD88, AKT2
Primary immunodeficiency	CIITA, RFX5, LCK
Measles	CCND3, FAS, PIK3R5, RACK1, IRF3, MYD88, AKT2

Bold: hub mRNAs.

**Table 3:** DEmiRNAs and DEmRNAs validated in GSE21321.

ID	RNA	*P* value	Log_2_(fold change)
17474	hsa-miR-421	0.054	0.042
10928	hsa-miR-125a-5p	0.018	0.123
21498	hsa-miR-654-3p	0.132	0.008
27542	hsa-miR-139-5p	0.553	0.007
27549	hsa-miR-548d-3p	0.094	0.017
29328	hsa-miR-582-3p	0.138	-0.006
42451	hsa-miR-139-3p	0.165	0.033
42470	hsa-miR-543	0.431	0.020
42750	hsa-miR-636	0.159	0.064
42811	hsa-miR-542-5p	0.142	0.014
42875	hsa-miR-330-5p	0.288	0.010
46355	hsa-miR-548p	0.184	-0.029
46625	hsa-miR-1303	0.134	-0.017
46705	hsa-miR-548k	0.124	-0.031
46752	hsa-miR-1270	0.283	-0.022
54850	FBXL12	0.001	-1.150
208	AKT2	0.016	-1.870
7335	UBE2V1	0.017	-2.820
1211	CLTA	0.037	0.056
960	CD44	0.034	0.698
51529	ANAPC11	0.176	-0.267
63891	RNF123	0.128	0.306
3932	LCK	0.136	-0.338
90933	TRIM41	0.152	-0.269
23533	PIK3R5	0.190	-0.403
22905	EPN2	0.408	-1.750
389	RHOC	0.856	-0.041
84727	SPSB2	0.620	0.258
142678	MIB2	0.924	0.020
64718	UNKL	0.847	0.090

## Data Availability

The data used in the paper is confidential. We cannot share the data underlying the findings of their manuscripts until the paper accepts it. Please contact us if you need the data.

## Supplementary Materials

Supplementary Materials Table 1: data filtering of circRNA, lncRNA, and mRNA. Table 2: summary of data cleaning from miRNA sequencing. Table 3: top 20 up- and downregulated lncRNAs in T2DM. Table 4: top 20 up- and downregulated circRNAs in T2DM. Table 5: top 20 up- and downregulated miRNAs in T2DM. Table 6: top 20 up- and downregulated mRNAs in T2DM. Table 7: the top 20 enriched KEGG pathways of the lncRNA-circRNA-miRNA-mRNA network in T2DM. Figure 1: the expression profiles of miRNAs. (A) The volcano plots of DEmiRNA. Red and green indicate up- and downregulation, respectively. (B) The cluster analysis (heatmaps) of DEmiRNA. The expression data was clustered with a log_10_(TPM + 1) value. The color scale indicates the expression of DEmiRNAs: red and blue indicate up- and downregulation, respectively. “T” represents the T2DM samples, and “C” represents the healthy controls. Figure 2: the expression profiles of mRNAs. (A) The volcano plots of DEmRNA. Red and green indicate up- and downregulation, respectively. (B) The cluster analysis (heatmaps) of DEmRNA. The expression data was clustered with a log_10_(TPM + 1) value. The color scale indicates the expression of DEmRNAs: red and blue indicate up- and downregulation, respectively. “T” represents the T2DM samples, and “C” represents the healthy controls. Figure 3: the chromosome map of DEmRNAs between the T2DM and control groups. “+”: chromosome positive chains; “-”: chromosome negative chains; the width of the bar represents the length of the RNA. Figure 4: the full ceRNA regulatory network. In this figure, lncRNA, circRNA, miRNA, and mRNA were indicated by diamond, rectangle, ellipse, and octagon, respectively. The node color changes gradually from green to red in ascending order according to the log_2_(fold change) of RNAs. Supplementary Figure 2: top 10 GO enrichment annotations: biological process (A), cellular component (B), and molecular function (C). The horizontal axis stands for the gene number which was enriched on the GO term and the vertical axis for the GO term name. The node color changed gradually from blue to red in ascending order according to the negative log_10_(*q*‐value).
