# Supplementary material for: High-Throughput Sequencing and Exploration of the lncRNA-circRNA-miRNA-mRNA Network in Type 2 Diabetes Mellitus
Source: Biomed Res Int. 2020 May 20;2020:8162524. doi: 10.1155/2020/8162524 (PMC7273392; doi:10.1155/2020/8162524)
Supplement: Supplementary Materials — Table 1: data filtering of circRNA, lncRNA, and mRNA. Table 2: summary of data cleaning from miRNA sequencing. Table 3: top 20 up- and downregulated lncRNAs in T2DM. Table 4: top 20 up- and downregulated circRNAs in T2DM. Table 5: top 20 up- and downregulated miRNAs in T2DM. Table 6: top 20 up- and downregulated mRNAs in T2DM. Table 7: the top 20 enriched KEGG pathways of the lncRNA-circRNA-miRNA-mRNA network in T2DM. Figure 1: the expression profiles of miRNAs. (A) The volcano plots of DEmiRNA. Red and green indicate up- and downregulation, respectively. (B) The cluster analysis (heatmaps) of DEmiRNA. The expression data was clustered with a log10(TPM + 1) value. The color scale indicates the expression of DEmiRNAs: red and blue indicate up- and downregulation, respectively. “T” represents the T2DM samples, and “C” represents the healthy controls. Figure 2: the expression profiles of mRNAs. (A) The volcano plots of DEmRNA. Red and green indicate up- and downregulation, respectively. (B) The cluster analysis (heatmaps) of DEmRNA. The expression data was clustered with a log10(TPM + 1) value. The color scale indicates the expression of DEmRNAs: red and blue indicate up- and downregulation, respectively. “T” represents the T2DM samples, and “C” represents the healthy controls. Figure 3: the chromosome map of DEmRNAs between the T2DM and control groups. “+”: chromosome positive chains; “-”: chromosome negative chains; the width of the bar represents the length of the RNA. Figure 4: the full ceRNA regulatory network. In this figure, lncRNA, circRNA, miRNA, and mRNA were indicated by diamond, rectangle, ellipse, and octagon, respectively. The node color changes gradually from green to red in ascending order according to the log2(fold change) of RNAs. Supplementary Figure 2: top 10 GO enrichment annotations: biological process (A), cellular component (B), and molecular function (C). The horizontal axis stands for the gene number which was enriched on the GO term and the v [file 8162524.f1.docx]

Supplementary:

| **Table 1 Data filtering of circRNA, lncRNA and mRNA** | | | | | | | |
| --- | --- | --- | --- | --- | --- | --- | --- |
| CircRNA | | | | | | | |
| sample | raw_reads | clean_reads | clean_bases | error_rate(%) | Q20(%) | Q30(%) | GC_content(%) |
| T1 | 82414560 | 79364942 | 11.9G | 0.02 | 96.97 | 92.57 | 58.33 |
| T2 | 91542470 | 87375680 | 13.11G | 0.02 | 96.19 | 90.84 | 59.76 |
| T3 | 106527102 | 103611692 | 15.54G | 0.02 | 97.24 | 92.92 | 61.26 |
| C1 | 97740922 | 97740922 | 14.66G | 0.02 | 97.27 | 92.93 | 61.65 |
| C2 | 97583408 | 97583408 | 14.64G | 0.01 | 97.85 | 94.18 | 60.5 |
| C3 | 100893034 | 100893034 | 15.13G | 0.01 | 97.77 | 94.11 | 57.96 |
| LncRNA | | | | | | | |
| T1 | 82414560 | 79364942 | 11.9G | 0.02 | 96.97 | 92.57 | 58.33 |
| T2 | 91542470 | 87375680 | 13.11G | 0.02 | 96.19 | 90.84 | 59.76 |
| T3 | 106527102 | 103611692 | 15.54G | 0.02 | 97.24 | 92.92 | 61.26 |
| C1 | 97740922 | 97740922 | 14.66G | 0.02 | 97.27 | 92.93 | 61.65 |
| C2 | 97583408 | 97583408 | 14.64G | 0.01 | 97.85 | 94.18 | 60.5 |
| C3 | 100893034 | 100893034 | 15.13G | 0.01 | 97.77 | 94.11 | 57.96 |
| mRNA | | | | | | | |
| T1 | 82414560 | 79364942 | 11.9G | 0.02 | 96.97 | 92.57 | 58.33 |
| T2 | 91542470 | 87375680 | 13.11G | 0.02 | 96.19 | 90.84 | 59.76 |
| T3 | 106527102 | 103611692 | 15.54G | 0.02 | 97.24 | 92.92 | 61.26 |
| C1 | 97740922 | 97740922 | 14.66G | 0.02 | 97.27 | 92.93 | 61.65 |
| C2 | 97583408 | 97583408 | 14.64G | 0.01 | 97.85 | 94.18 | 60.5 |
| C3 | 100893034 | 100893034 | 15.13G | 0.01 | 97.77 | 94.11 | 57.96 |

| **Table 2 Summary of data cleaning from miRNA sequencing** | | | | | | | |
| --- | --- | --- | --- | --- | --- | --- | --- |
| Sample | Total reads | N%>10% | low quality | 5 adapter  contamine | 3 adapter null or insert null | with  ployA/T/G/C | clean reads |
| T1 | 12778191 (100.00%) | 0 (0.00%) | 122139 (0.96%) | 391 (0.00%) | 146306 (1.14%) | 1526 (0.01%) | 12507829 (97.88%) |
| T2 | 13107612 (100.00%) | 0 (0.00%) | 137294 (1.05%) | 384 (0.00%) | 150230 (1.15%) | 1990 (0.02%) | 12817714 (97.79%) |
| T3 | 11677326 (100.00%) | 0 (0.00%) | 126144 (1.08%) | 742 (0.01%) | 135162 (1.16%) | 2285 (0.02%) | 11412993 (97.74%) |
| C1 | 12476427 (100.00%) | 0 (0.00%) | 86664 (0.69%) | 128 (0.00%) | 290145 (2.33%) | 2430 (0.02%) | 12097060 (96.96%) |
| C2 | 12165981 (100.00%) | 6 (0.00%) | 97346 (0.80%) | 270 (0.00%) | 346923 (2.85%) | 2561 (0.02%) | 11718875 (96.32%) |
| C3 | 14285486 (100.00%) | 113 (0.00%) | 110281 (0.77%) | 206 (0.00%) | 352397 (2.47%) | 3845 (0.03%) | 13818644 (96.73%) |

| **Table 3 Top 20 up- and down-regulated lncRNAs in T2DM** | | | |
| --- | --- | --- | --- |
| lncRNA | Log_2_(Fold change) | *P*-value | Regulation |
| LNC_001633 | Inf | 9.48E-07 | up |
| LNC_000275 | 8.856999007 | 1.28E-06 | up |
| LNC_002636 | 6.653615852 | 2.57E-06 | up |
| LNC_002407 | Inf | 2.94E-05 | up |
| LNC_000262 | 7.38576939 | 3.70E-05 | up |
| LNC_001662 | Inf | 7.37E-05 | up |
| LNC_002672 | 6.321721311 | 0.000152456 | up |
| ENST00000616469.4 | Inf | 0.00017606 | up |
| LNC_001726 | Inf | 0.000186761 | up |
| LNC_000117 | 4.721708494 | 0.000198596 | up |
| LNC_002167 | Inf | 0.000240254 | up |
| LNC_000644 | Inf | 0.000362219 | up |
| LNC_002063 | 8.123754342 | 0.000418453 | up |
| LNC_000100 | Inf | 0.000512593 | up |
| LNC_000633 | Inf | 0.000544115 | up |
| LNC_000817 | 4.918039792 | 0.000592496 | up |
| LNC_002277 | Inf | 0.000702766 | up |
| LNC_001776 | Inf | 0.000846204 | up |
| LNC_000357 | Inf | 0.000892751 | up |
| LNC_000804 | Inf | 0.000998506 | up |
| LNC_002793 | -Inf | 3.28E-05 | down |
| LNC_001252 | -Inf | 0.000312577 | down |
| LNC_000017 | -Inf | 0.000568781 | down |
| LNC_001556 | -Inf | 0.000604376 | down |
| LNC_002794 | -Inf | 0.000838452 | down |
| LNC_000016 | -Inf | 0.000882833 | down |
| LNC_001558 | -4.403676573 | 0.001280385 | down |
| LNC_001129 | -Inf | 0.001473499 | down |
| LNC_001475 | -Inf | 0.001662023 | down |
| LNC_001908 | -7.040210889 | 0.002474714 | down |
| LNC_001912 | -4.918185036 | 0.003273191 | down |
| LNC_001560 | -5.275553173 | 0.003797249 | down |
| ENST00000533146.5 | -Inf | 0.004353141 | down |
| LNC_001640 | -Inf | 0.00484157 | down |
| LNC_002487 | -Inf | 0.005184552 | down |
| ENST00000567093.1 | -3.104338864 | 0.005366581 | down |
| LNC_001616 | -Inf | 0.005495317 | down |
| LNC_000074 | -Inf | 0.006672802 | down |
| LNC_000541 | -4.71116752 | 0.006926685 | down |
| ENST00000592918.5 | -2.750591507 | 0.007488405 | down |
| Inf: maximum (max); -Inf: minimum | | | |

| **Table 4 Top 20 up- and down-regulated circRNAs in T2DM** | | | |
| --- | --- | --- | --- |
| circRNA | Log_2_(Fold change) | *P*-value | Regulation |
| novel_circ_0002486 | 4.3253 | 2.13E-15 | up |
| hsa_circ_0002661 | 3.8647 | 3.67E-07 | up |
| hsa_circ_0002590 | 3.5141 | 3.94E-06 | up |
| hsa_circ_0046580 | 3.4841 | 7.46E-06 | up |
| hsa_circ_0088567 | 3.309 | 2.64E-05 | up |
| novel_circ_0026461 | 3.2827 | 2.81E-05 | up |
| novel_circ_0002455 | 3.2081 | 4.88E-05 | up |
| hsa_circ_0053394 | 3.1948 | 5.37E-05 | up |
| novel_circ_0021094 | 3.0742 | 0.00010757 | up |
| hsa_circ_0002479 | 3.0237 | 0.00013935 | up |
| hsa_circ_0007083 | 2.7444 | 0.00015882 | up |
| hsa_circ_0047378 | 2.9833 | 0.00018016 | up |
| hsa_circ_0005035 | 2.7524 | 0.00018176 | up |
| novel_circ_0028566 | 2.9429 | 0.00021997 | up |
| novel_circ_0025666 | 2.9318 | 0.00023555 | up |
| novel_circ_0022773 | 2.8971 | 0.00028605 | up |
| novel_circ_0002620 | 2.8546 | 0.0003583 | up |
| novel_circ_0002621 | 2.8436 | 0.00037921 | up |
| hsa_circ_0086694 | -4.7216 | 3.36E-13 | down |
| hsa_circ_0075723 | -5.169 | 5.33E-13 | down |
| novel_circ_0014395 | -5.1217 | 7.44E-13 | down |
| hsa_circ_0001851 | -5.1234 | 7.98E-13 | down |
| novel_circ_0028458 | -4.2439 | 1.63E-08 | down |
| hsa_circ_0000798 | -3.3024 | 1.14E-07 | down |
| hsa_circ_0005718 | -3.1508 | 2.94E-07 | down |
| hsa_circ_0006291 | -3.8435 | 5.12E-07 | down |
| novel_circ_0001727 | -3.2373 | 6.71E-07 | down |
| novel_circ_0002353 | -3.5943 | 3.64E-06 | down |
| novel_circ_0027247 | -2.9921 | 4.07E-06 | down |
| novel_circ_0011759 | -3.1098 | 4.36E-06 | down |
| hsa_circ_0036865 | -3.2532 | 3.77E-05 | down |
| hsa_circ_0004856 | -3.2269 | 4.43E-05 | down |
| hsa_circ_0023558 | -1.711 | 4.57E-05 | down |
| hsa_circ_0058275 | -2.3964 | 5.44E-05 | down |
| novel_circ_0002246 | -3.1894 | 6.04E-05 | down |
| novel_circ_0021265 | -2.0358 | 6.06E-05 | down |
| novel_circ_0009843 | -3.1683 | 6.25E-05 | down |
| novel_circ_0027471 | -3.1567 | 6.91E-05 | down |

| **Table 5 Top 20 up- and down-regulated miRNAs in T2DM** | | | |
| --- | --- | --- | --- |
| miRNA | Log_2_(Fold change) | *P*-value | Regulation |
| hsa-miR-4473 | 3.2006 | 1.63E-25 | up |
| hsa-miR-363-3p | 1.6842 | 5.38E-22 | up |
| hsa-miR-548o-3p | 2.0393 | 1.10E-21 | up |
| hsa-miR-151a-3p | 3.0015 | 2.52E-19 | up |
| hsa-miR-3909 | 1.8095 | 9.68E-18 | up |
| hsa-miR-99a-5p | 1.5613 | 4.36E-17 | up |
| hsa-miR-11401 | 1.7729 | 1.04E-16 | up |
| hsa-miR-132-5p | 1.9837 | 1.81E-16 | up |
| hsa-miR-29c-5p | 2.8248 | 9.85E-16 | up |
| hsa-miR-139-5p | 2.7918 | 1.68E-15 | up |
| hsa-miR-340-3p | 2.5821 | 2.74E-15 | up |
| hsa-miR-148a-3p | 3.1326 | 5.73E-15 | up |
| hsa-miR-548ah-3p | 2.1013 | 1.02E-14 | up |
| hsa-miR-6806-3p | 2.7946 | 1.13E-14 | up |
| hsa-miR-3158-3p | 3.4493 | 2.05E-13 | up |
| hsa-miR-3158-5p | 3.4491 | 2.08E-13 | up |
| hsa-miR-126-3p | 2.1006 | 2.86E-13 | up |
| hsa-miR-330-5p | 2.3573 | 4.40E-13 | up |
| hsa-miR-6842-3p | 2.597 | 1.38E-12 | up |
| hsa-miR-548am-3p | 1.8586 | 1.38E-12 | up |
| hsa-miR-421 | -2.431 | 5.01E-22 | down |
| hsa-miR-16-5p | -1.9284 | 1.47E-17 | down |
| hsa-miR-106b-5p | -1.7941 | 1.62E-16 | down |
| hsa-miR-548k | -1.9443 | 2.44E-14 | down |
| hsa-miR-660-5p | -1.9429 | 1.03E-13 | down |
| hsa-miR-550a-3p | -2.323 | 4.35E-13 | down |
| hsa-miR-550b-2-5p | -2.323 | 4.35E-13 | down |
| hsa-miR-6511a-3p | -1.9976 | 3.16E-12 | down |
| hsa-miR-15a-5p | -1.7596 | 6.86E-12 | down |
| hsa-miR-29b-3p | -1.6668 | 2.86E-11 | down |
| hsa-miR-191-5p | -1.6063 | 2.42E-08 | down |
| hsa-miR-29c-3p | -1.8601 | 3.51E-08 | down |
| hsa-miR-454-5p | -1.7687 | 1.32E-07 | down |
| hsa-miR-20a-5p | -1.7449 | 1.63E-07 | down |
| hsa-miR-125a-5p | -1.7256 | 3.75E-07 | down |
| hsa-miR-190a-5p | -1.6091 | 3.92E-07 | down |
| hsa-miR-126-5p | -2.1131 | 5.43E-07 | down |
| hsa-miR-30b-5p | -1.9072 | 3.24E-06 | down |
| hsa-miR-1271-5p | -2.2054 | 4.10E-06 | down |
| hsa-miR-29b-2-5p | -1.5121 | 5.89E-06 | down |

| **Table 6 Top 20 up- and down-regulated mRNAs in T2DM** | | | |
| --- | --- | --- | --- |
| mRNA | Log_2_(Fold change) | *P*-value | Regulation |
| SBNO2 | Inf | 4.34E-15 | up |
| TPGS2 | Inf | 1.33E-12 | up |
| FCHO1 | Inf | 3.30E-11 | up |
| POLD1 | Inf | 9.43E-10 | up |
| CFL1 | Inf | 1.06E-09 | up |
| SLC11A2 | Inf | 3.37E-09 | up |
| ALS2CR12 | 9.679369916 | 1.25E-08 | up |
| SMC1A | 8.513524614 | 1.35E-08 | up |
| SMURF1 | 10.21403372 | 5.59E-08 | up |
| HMOX2 | 7.33482772 | 2.31E-07 | up |
| SCARF1 | Inf | 2.45E-07 | up |
| ECSIT | Inf | 3.27E-07 | up |
| PLEKHB2 | Inf | 6.26E-07 | up |
| RPLP0 | Inf | 8.75E-07 | up |
| CTSD | Inf | 1.19E-06 | up |
| ABHD2 | Inf | 2.36E-06 | up |
| TBC1D17 | 6.045930961 | 3.85E-06 | up |
| ZMYND8 | Inf | 3.89E-06 | up |
| TAB2 | 6.08090496 | 3.94E-06 | up |
| VAV1 | Inf | 4.25E-06 | up |
| ZMYND8 | -Inf | 8.49E-13 | down |
| NSD1 | -Inf | 9.69E-12 | down |
| SIPA1L1 | -Inf | 9.76E-12 | down |
| MT-ATP8 | -7.989257424 | 1.87E-11 | down |
| TLK1 | -Inf | 4.12E-11 | down |
| IKZF1 | -Inf | 6.52E-11 | down |
| FOXP1 | -Inf | 2.99E-10 | down |
| SPECC1 | -Inf | 7.67E-10 | down |
| ZNF385A | -10.50403435 | 8.23E-10 | down |
| USP8 | -Inf | 2.26E-09 | down |
| ATM | -Inf | 4.46E-09 | down |
| ATXN2L | -Inf | 6.64E-09 | down |
| LRRC37A | -Inf | 8.02E-09 | down |
| UBE2K | -Inf | 8.13E-09 | down |
| PPP6R3 | -9.934343518 | 8.85E-09 | down |
| PUF60 | -Inf | 1.19E-08 | down |
| PUF60 | -Inf | 1.98E-08 | down |
| ZMYND8 | -9.498486909 | 2.22E-08 | down |
| UBAP2L | -Inf | 3.20E-08 | down |
| EP400 | -Inf | 3.39E-08 | down |
| Inf: maximum (max); -Inf: minimum | | | |

| **Table 7 The top 20 enriched KEGG pathways of lncRNA-circRNA-miRNA-mRNA network in T2DM** | | | | | |
| --- | --- | --- | --- | --- | --- |
| KEGG pathway | Pathway  ID | Risk  factor | Gene  number | Background  number | *P*adj-value |
| Fc gamma R-mediated phagocytosis | hsa04666 | 0.077 | 7 | 91 | 0.443 |
| Fanconi anemia pathway | hsa03460 | 0.094 | 5 | 53 | 0.443 |
| Tuberculosis | hsa05152 | 0.050 | 9 | 179 | 0.470 |
| Glycerophospholipid metabolism | hsa00564 | 0.065 | 6 | 92 | 0.470 |
| Histidine metabolism | hsa00340 | 0.130 | 3 | 23 | 0.470 |
| Collecting duct acid secretion | hsa04966 | 0.111 | 3 | 27 | 0.579 |
| Base excision repair | hsa03410 | 0.091 | 3 | 33 | 0.643 |
| mTOR signaling pathway | hsa04150 | 0.067 | 4 | 60 | 0.643 |
| Lysosome | hsa04142 | 0.049 | 6 | 122 | 0.643 |
| mRNA surveillance pathway | hsa03015 | 0.055 | 5 | 91 | 0.643 |
| Synaptic vesicle cycle | hsa04721 | 0.063 | 4 | 63 | 0.643 |
| p53 signaling pathway | hsa04115 | 0.059 | 4 | 68 | 0.737 |
| Other glycan degradation | hsa00511 | 0.111 | 2 | 18 | 0.746 |
| T cell receptor signaling pathway | hsa04660 | 0.048 | 5 | 104 | 0.746 |
| HTLV-I infection | hsa05166 | 0.034 | 9 | 261 | 0.802 |
| Mismatch repair | hsa03430 | 0.087 | 2 | 23 | 0.802 |
| Endometrial cancer | hsa05213 | 0.058 | 3 | 52 | 0.802 |
| Phagosome | hsa04145 | 0.039 | 6 | 155 | 0.802 |
| Glycosylphosphatidylinositol(GPI)-anchor biosynthesis | hsa00563 | 0.080 | 2 | 25 | 0.802 |
| Vibrio cholerae infection | hsa05110 | 0.056 | 3 | 54 | 0.802 |


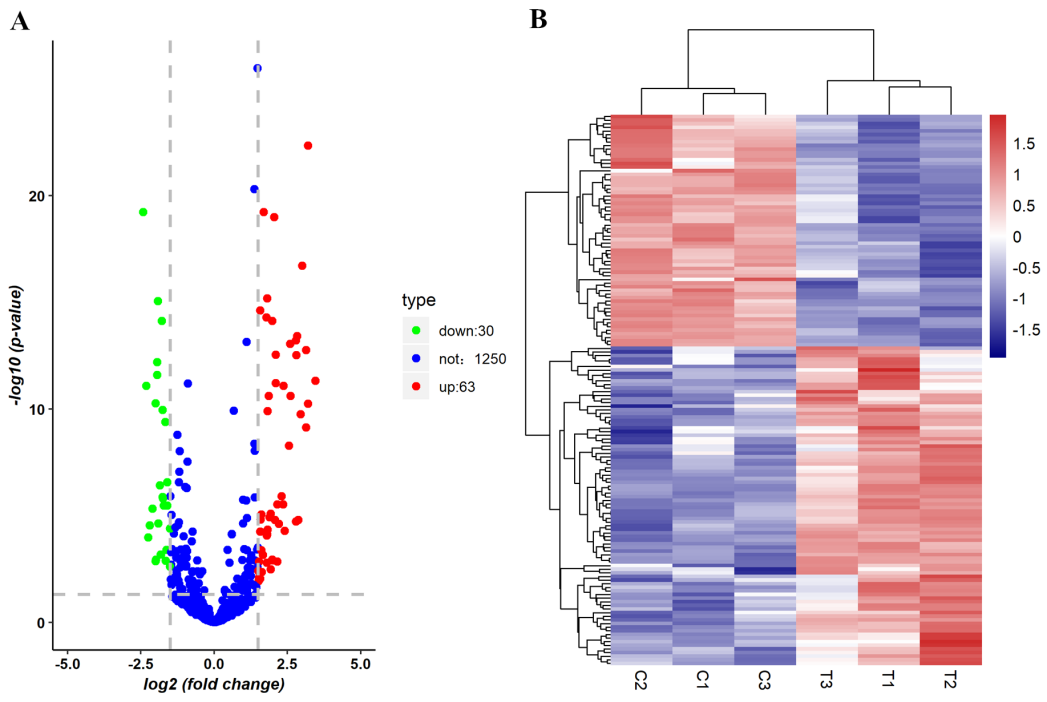


**Fig. 1** **The expression profiles of miRNAs.** (A) The volcano plots of DEmiRNA. Red, and green indicate up- and down-regulation, respectively. (B) The cluster analysis (heatmaps) of DEmiRNA. The expression data was clustered with log_10_ (TPM+1) value. The color scale indicates the expression of DEmiRNAs: red and blue indicate up- and down-regulation, respectively. ‘‘T’’ represents the T2DM samples, and ‘‘C’’ represents the healthy controls.


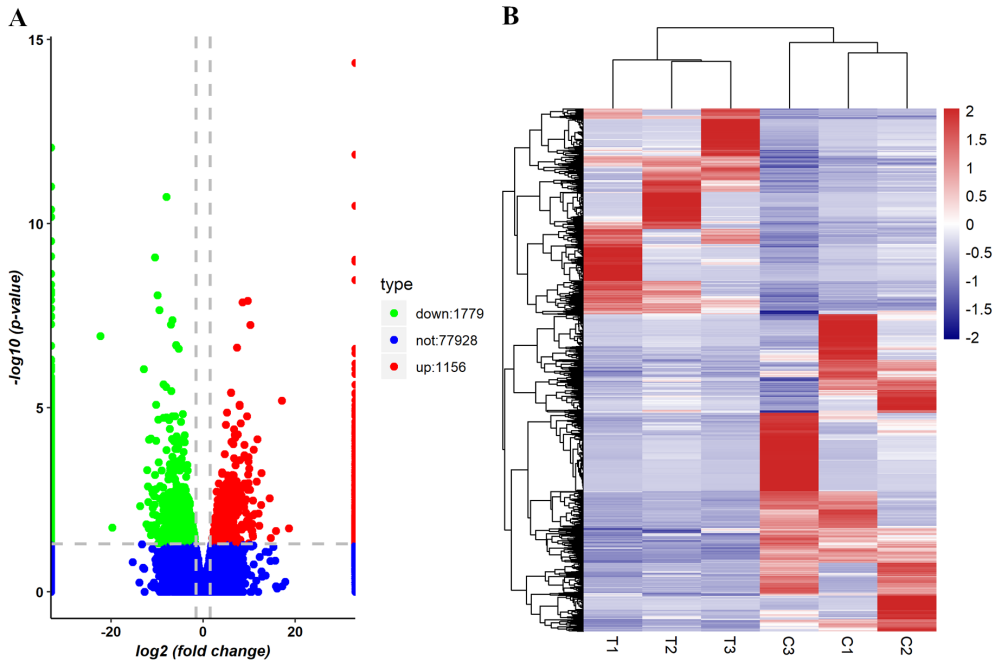


**Fig. 2** **The expression profiles of mRNAs.** (A) The volcano plots of DEmRNA. Red, and green indicate up- and down-regulation, respectively. (B) The cluster analysis (heatmaps) of DEmRNA. The expression data was clustered with log_10_ (TPM+1) value. The color scale indicates the expression of DCEmRNAs: red and blue indicate up- and down-regulation, respectively. ‘‘T’’ represents the T2DM samples, and ‘‘C’’ represents the healthy controls.


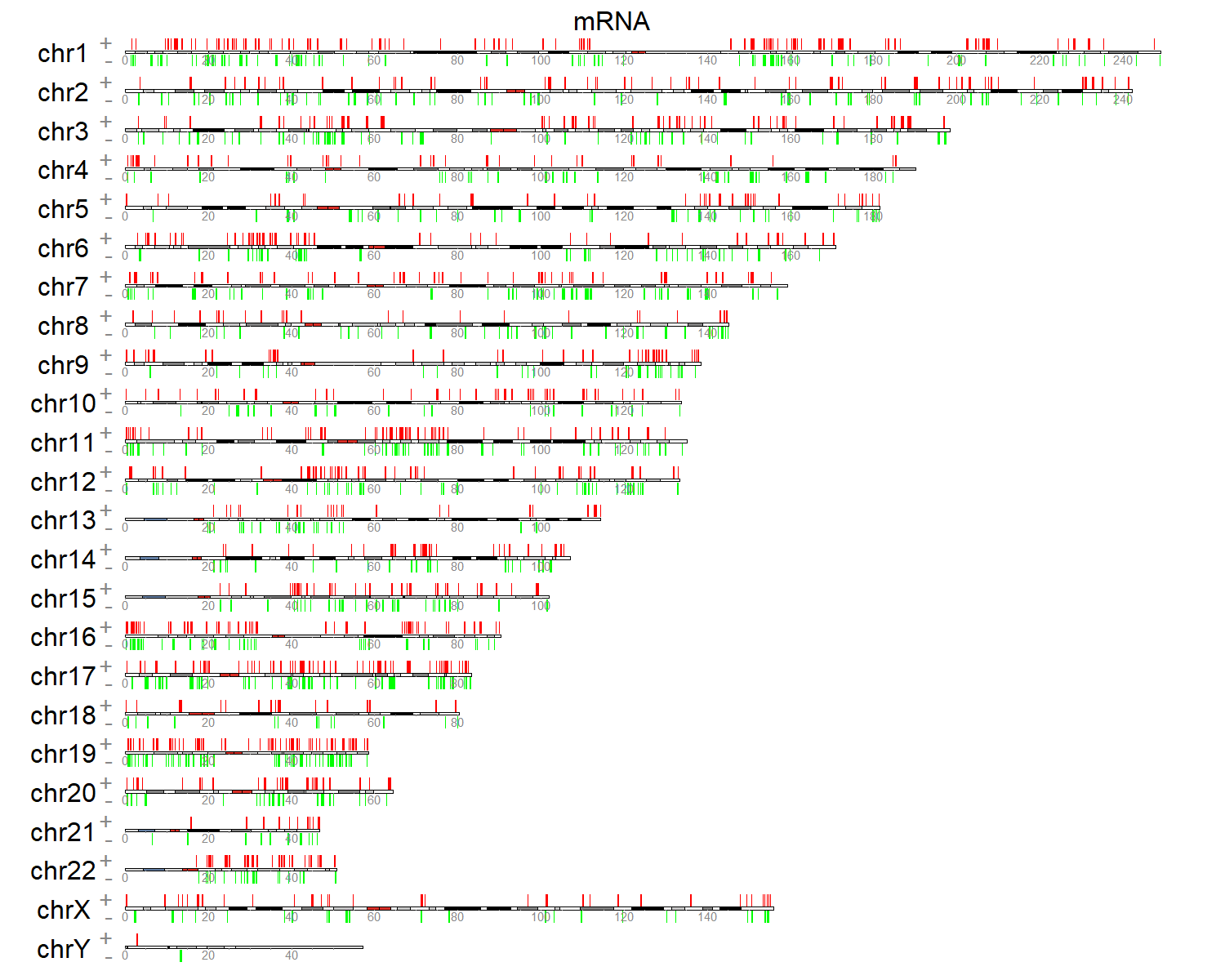


**Fig. 3** **The** **chromosome map of DEmRNAs between T2DM and control group.** “+”: chromosome positive chains, “-”: chromosome negative chains; the width of the bar represents the length of the RNA.


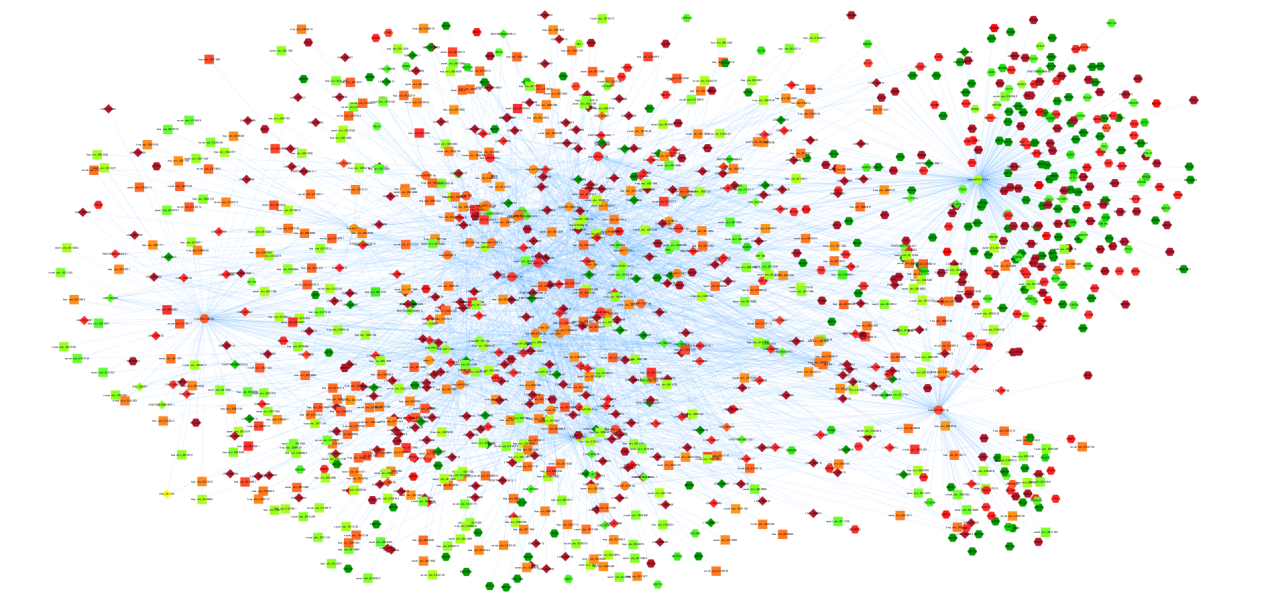


**Fig. 4** **The full ceRNA regulatory network.** In this figure, lncRNA, circRNA, miRNA, and mRNA were indicated to diamond, rectangle, ellipse and octagon, respectively. The node color changes gradually from green to red in ascending order according to the log2(foldchange) of RNAs.


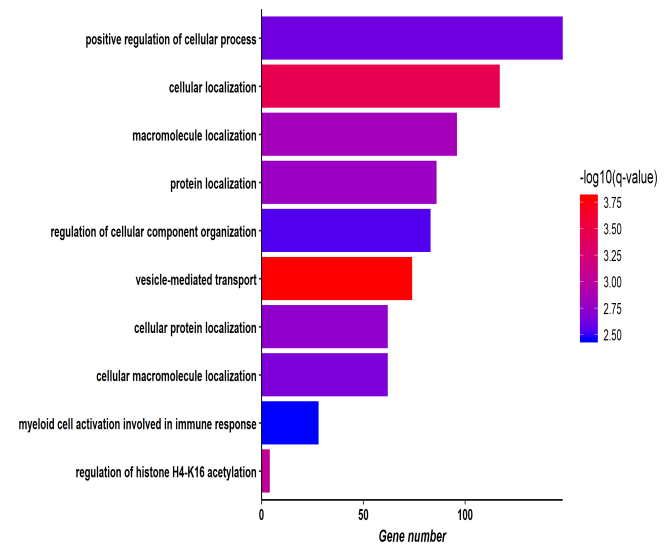

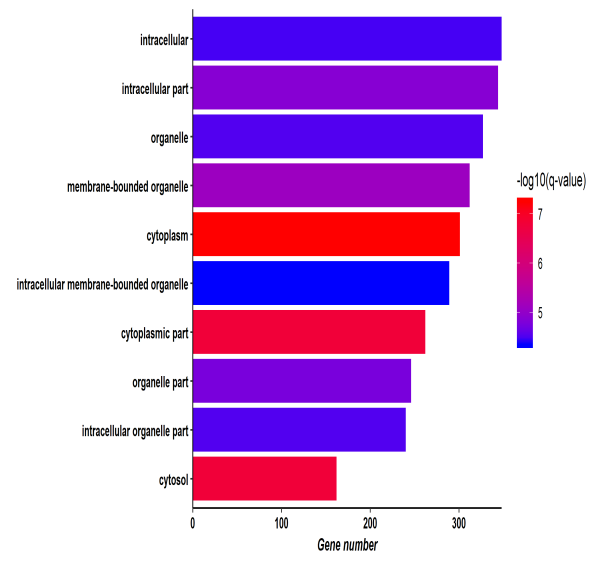


(B)

(A)


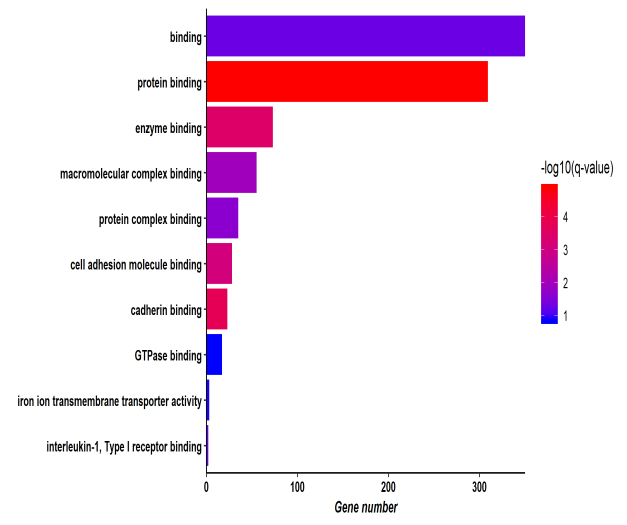


(C)

**Supplementary Figure 2. Top 10 GO enrichment annotations: biological process (A); cellular component (B); molecular function (C).** The horizontal axis standed for the gene number which enriched on the GO term, vertical axis for the GO term name. The node color changed gradually from blue to red in ascending order according to the negatived log10 (q-value).
